# Comparison of Total Hip Arthroplasty Outcomes after Failed Femoral Wedge or Curved Varus Osteotomy

**DOI:** 10.2174/1874325001812010208

**Published:** 2018-06-25

**Authors:** Motoki Sonohata, Masaru Kitajima, Shunsuke Kawano, Masaaki Mawatari

**Affiliations:** Department of Orthopaedic Surgery, Faculty of Medicine, Saga University 5-1-1 Nabeshima, Saga 849-8501, Japan

**Keywords:** Total hip arthroplasty, Varus osteotomy, Curved varus osteotomy, Wedge varus osteotomy, Follow-up studies, Subtrochanteric corrective osteotomy, Clinical outcomes, Radiographic outcomes

## Abstract

**Background:**

Differences in clinical and radiographic results following total hip arthroplasty between failed wedge and curved varus osteotomy are unclear.

**Objective:**

To investigate differences in clinical and radiographic results following total hip arthroplasty in patients who exhibited failed wedge or curved varus osteotomy.

**Method:**

We performed 18 total hip arthroplasties after failed femoral varus osteotomy. Hips were divided into two groups: 14 had failed wedge varus osteotomy and four had failed curved varus osteotomy. Average ages at osteotomy and total hip arthroplasty were 27 years old (range, 10-46 years old) and 56 years old (range, 25-74 years old), respectively. The average duration of follow-up monitoring was 72.2 months (range, 54-91 months). Clinical and radiographic evaluations were completed for each group.

**Results:**

The Japanese Orthopaedic Association hip score of total hip arthroplasty after failed varus osteotomy significantly improved at the last follow-up in both groups. However, hip score at the last follow-up was significantly higher after failed curved varus osteotomy than after failed wedge varus osteotomy (p<0.01). Four hips that failed wedge varus osteotomy underwent subtrochanteric corrective osteotomy with total hip arthroplasty. Radiographic evaluation showed that three stems for total hip arthroplasty after failed wedge varus osteotomy were inserted in malposition, and all stems in total hip arthroplasty after failed curved varus osteotomy were inserted in the normal position.

**Conclusion:**

Surgeons performing femoral varus osteotomy should consider possible future conversion to total hip arthroplasty. Curved varus osteotomy is more suitable than wedge varus osteotomy for future conversion to total hip arthroplasty.

## 1 INTRODUCTION

Femoral varus osteotomy is a method to manage pre-arthritic and early arthritic hip conditions, including primary osteoarthritis, secondary osteoarthritis due to developmental dysplasia of the hip joint, and avascular necrosis; these can be challenging due to the relatively young patient age at presentation, high variation in structural deformities, and increased risk of secondary hip osteoarthritis over time [1-8].

Some patients experience a change in their hip disease after osteotomy, and several lines of evidence indicate good clinical results following femoral varus osteotomy [1-8]. However, femoral varus osteotomy does not always enable full recovery; some patients require further treatment with Total Hip Arthroplasty (THA) for hip osteoarthritis following femoral varus osteotomy. Previous studies have reported success rates for femoral varus osteotomy of 73%-90% [7, 9].

THA for patients with a history of femoral osteotomy is technically difficult, and there are more complications associated with this procedure than with traditional THA. Furthermore, THA performed after intertrochanteric osteotomy for osteoarthritis is known to have higher perioperative and postoperative complication rates (17%-25%) than THA in patients without a history of osteotomy [10, 11]. Femoral varus osteotomy procedures have been described previously [1, 12] and can be divided into two groups: curved and wedge varus osteotomy. Curved varus osteotomy was first described by Nishio and Sugioka [1]; this procedure retains leg length after the operation and protects against Trendelenburg’s sign. There have been a few reports of THA after failed femoral varus osteotomy [13]; however, differences in clinical and radiographic results between patients who failed curved varus osteotomy and those who failed wedge varus osteotomy are unknown.

We herein report clinical and radiographic outcomes of THA after failed femoral varus osteotomy, comparing clinical and radiographic outcomes between patients who had failed curved varus osteotomy and those who had failed wedge varus osteotomy.

## 2 MATERIAL AND METHODS

The study protocol adhered to the ethical guidelines of the 1975 Declaration of Helsinki, and the institutional review board approved this study. Written informed consent was obtained from all participants.

Between February 1999 and December 2011, we performed cementless THA in 22 hips (19 patients) after failed intertrochanteric varus osteotomy. All varus osteotomy procedures were performed at other hospitals, except for one hip. We excluded four hips (four patients) that we were unable to monitor for more than 4 years after THA. We enrolled the remaining 18 hips in 15 patients in the present study.

**Fig. (1):**
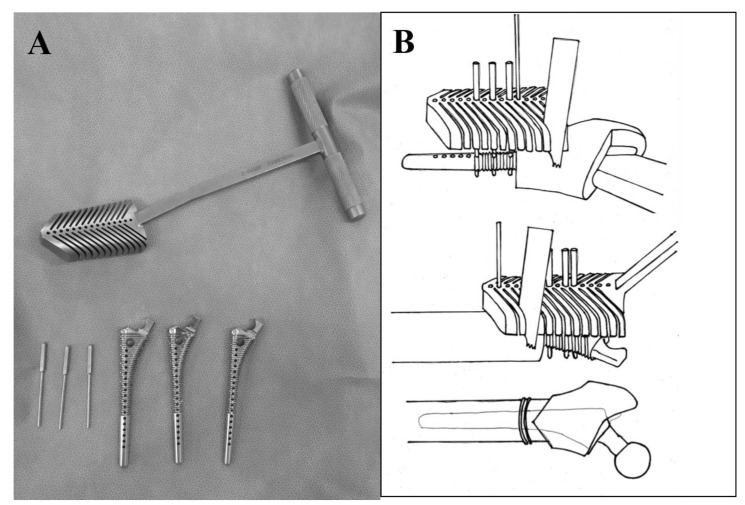
V-shaped subtrochanteric osteotomy device and its usage. **A**: Photograph of the device. **B**: Diagrams showing V-shaped subtrochanteric osteotomy.

We adopted the posterolateral approach in all cases, due to the advantages of using an extensive approach for the greater trochanter and proximal femur. All patients received only spinal anesthesia. Fourteen hips underwent conventional THA, and four hips underwent THA with subtrochanteric corrective osteotomy. In three hips, V-shaped subtrochanteric corrective osteotomy was performed by using a device to help ensure the performance of an accurate osteotomy based on the shape of the femur medullary cavity [14] (Fig. **1**). In one hip, transverse subtrochanteric corrective osteotomy was performed, because the corrective osteotomy was excessively proximal for V-shaped osteotomy. The decision to perform subtrochanteric corrective osteotomy was made by the operator. Following conventional THA, all patients were allowed full weight-bearing 2 days after the operation. Following THA with subtrochanteric corrective osteotomy, patients were allowed to use a wheelchair 2 days after the operation; partial weight-bearing with crutches or a walker was allowed 1 week after the operation, while standing and walking with full weight-bearing was allowed 3 weeks after the operation.

All of the operations were performed by using a cementless femoral component (a PerFix-HA femoral component; Kyocera, Kyoto, Japan) with a 28- or 32-mm zirconia ball and an AMS-HA acetabular shell (Kyocera, Kyoto, Japan) with an AMS (cross-linked ultra-high-molecular-weight polyethylene liner) liner (Kyocera, Kyoto, Japan). All hips were evaluated by using the Japanese Orthopaedic Association (JOA) hip score before THA and last follow-up. JOA hip score consisted of four categories, with 100 points regarded as full marks: pain (40 points), range of motion (20 points), walking ability (20 points), and activities of daily living (20 points).

Routine radiographic examinations included anteroposterior and frog-leg lateral radiographs. Acetabular components were evaluated at the most recent follow-up for evidence of migration, in accordance with the method of Carlsson and Gentz [15]. The bone-metal interface was evaluated at the most recent follow-up for the presence and progression of radiolucent lines in the three zones described by DeLee and Charnley [16]. The femoral component was evaluated for changes in position, subsidence, and radiolucencies in the seven zones described by Gruen *et al*. [17]. Stability of the femoral component was assessed as bone-ingrown fixation, stable fixation, or unstable fixation, in accordance with the fixation/stability score described by Engh *et al*. [18]. The grade of dislocation was evaluated by using Crowe’s classification [19].

Abduction and anteversion angles of the acetabular components and alignments of the femoral stems were measured on the most recent anteroposterior radiographs. The abduction angle of the acetabular component was measured by using the method described by Engh *et al*. [20, 21]. Anteversion of the acetabular component was calculated by using the method of Widmer [22]. Cups with an abduction angle of ≤30° or ≥50° [23], or with an anteversion angle of ≤5° or ≥25° [24], were considered outliers of optimal cup position. Stem alignment was determined by measuring the angle formed between the longitudinal axis of the femoral stem and the longitudinal axis of the femoral canal [25, 26]. The alignment of the stem was classified as neutral, valgus (>5° of lateral deviation), or varus (>5° of medial deviation) [25, 26].

**Fig. (2):**
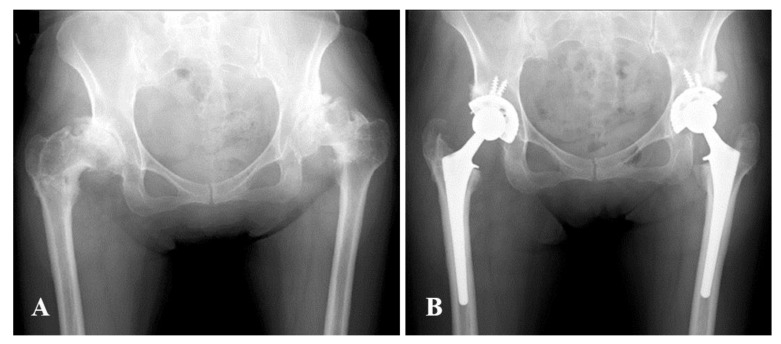
Pre- and postoperative radiographs of the bilateral hip joint of a 53-year-old woman. (**A**) After failed bilateral curved varus osteotomy of the bilateral femur. (**B**) Eighty-one months after total hip arthroplasty.

**Fig. (3):**
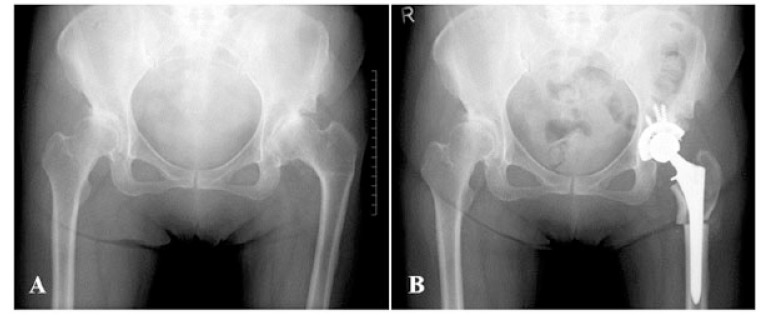
Pre- and postoperative radiographs of the left hip joint of a 60-year-old woman. (**A**) After failed left wedge varus osteotomy of the left femur. (**B**) Fifty-five months after total hip arthroplasty combined with subtrochanteric corrective femoral osteotomy.

Subjects were divided into two groups: hips that underwent curved varus osteotomy (Curved group) (Fig. **2**), and hips that underwent wedge varus osteotomy (Wedge group) (Fig. **3**). We compared the above data between the two groups.

Statistical Package for Social Sciences (SPSS) version 19 software program (IBM SPSS, Chicago, IL, USA) was used for statistical analyses of data. An unpaired *t*-test and the χ^2^ test were used to compare each of the parameters between the groups. A paired *t*-test was used to compare each of the parameters before and after THA. P values < 0.05 were considered to indicate statistical significance.

## 3 RESULTS

### 3.1 Clinical Evaluation

Regarding the patients’ background, there were no significant differences between the two groups (Table **1**). We noted no significant differences between the two groups in terms of perioperative results; however, all four femoral subtrochanteric corrective osteotomies combined with THA were performed in the Wedge group (Table **2**). There were 13 females and two males, and the average age at the time of the operation was 56 years old (range, 25-74 years old). The average duration of follow-up monitoring was 72.2 months (range, 54-91 months). Indications for the procedure were severe hip pain and/or considerable difficulty walking and performing daily activities. Mean duration between osteotomy and THA was 29.1 years (range, 11-51 years). In five hips, remaining implants were observed on radiographs before the operation (four plates and one screw). Chiari osteotomies were performed at the pelvic site in two hips.

Average total JOA hip score for all patients improved from 45.1 (range 26-77) preoperatively to 85.1 (range 62-96) at the latest follow-up (p<0.01). There was no statistically significant difference in each category; however, pain score was greatly improved (in all subjects and in a subgroup of subjects that excluded subtrochanteric osteotomy cases). After excluding four cases treated with femoral subtrochanteric corrective osteotomy, average total JOA hip score improved from 48.9 to 87.1 (p<0.01). Before THA, there were no significant differences between the two groups in average total JOA hip score or any of the four subcategories. However, at the latest follow-up, we noted significant differences between the groups in average total JOA hip score (p<0.01) and one subcategory (gait) (p<0.01) (Table **3**). Even after excluding four cases treated with femoral subtrochanteric corrective osteotomy, significant differences remained between the two groups in average total JOA hip score and gait subcategory at the latest follow-up. The average total JOA hip scores and gait subcategory values (Wedge group *vs*. Curved group) were 84.4 *vs*. 94.0 and 15.7 *vs*. 20.0, respectively. There were significant differences (p<0.01 and p<0.01, respectively). Regarding complications, there was one case of dislocation and transient paresthesia in one patient, who was in the Wedge group and underwent 3.7-cm leg lengthening. There were no cases of infection.

### 3.2 Radiographic Evaluation

**Fig. (4):**
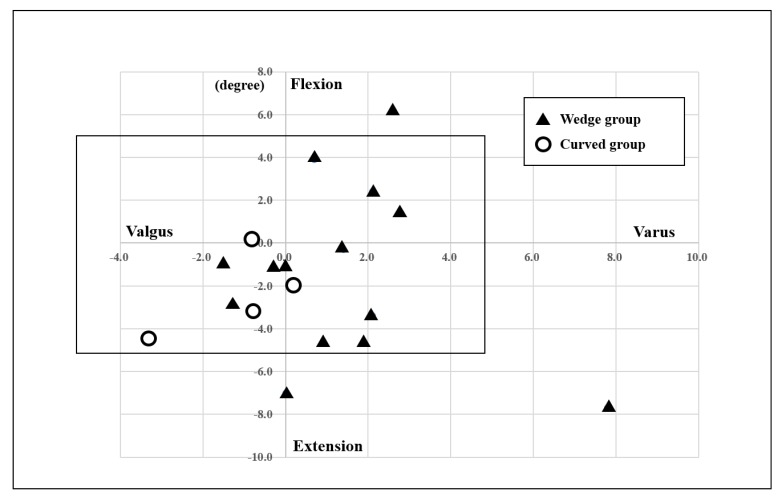
Stem alignment in both groups. All stems in malposition belong to the Wedge group.

No acetabular components showed any evidence of migration, loosening, or radiolucent lines of <2-mm thickness. One femoral component used in a case of femoral subtrochanteric corrective osteotomy moved toward the varus direction postoperatively; it was fixed with an optimum interface at the latest follow-up. Other femoral components were also fixed with an optimum interface at the latest follow-up. Bony union at the osteotomy site was achieved in all cases with subtrochanteric osteotomy. There were significant differences in cup abduction between the two groups; the average degree of cup anteversion in the Wedge group was significantly larger than that in the Curved group (P<0.05). There were no significant differences between the two groups regarding the number of cups in malposition (degree of abduction and anteversion), the number of stems in malposition (varus-valgus), or the number of stems in malposition (flexion-extension) (Table **4**). However, all stems of three hips in malposition belonged to the Wedge group (Fig. **4**); two hips exhibited Crowe grade 1 and one hip exhibited grade 3. One stem in malposition (varus-valgus) was observed in a hip that underwent femoral subtrochanteric corrective osteotomy. Two stems in malposition (flexion-extension) were observed in hips that underwent femoral subtrochanteric corrective osteotomy. There were no cases of revision at the last follow-up.

## 4 DISCUSSION

There have been several reports regarding outcomes of THA after failed femoral osteotomy, including valgus osteotomy, anterior rotational osteotomy, and Schanz osteotomy [26-28]. However, few studies have described outcomes of THA after failed femoral varus osteotomy; thus far, only one study has examined the results of conversion THA after curved varus osteotomy [13]. Takegami *et al*. reported that THA after failed curved varus osteotomy provides satisfactory clinical outcomes; Harris hip score significantly improved from 53.8 to 89.7 [13].

There are two types of varus osteotomy: curved [1] and wedge [12] osteotomy. Curved osteotomy has an advantage over wedged varus osteotomy in the degree of leg length discrepancy after the operation [8]. However, good clinical results have been reported after both curved and wedge varus osteotomy procedures. Okura *et al*. [7] reported a 90% survival rate at 10-year follow-up of femoral curved osteotomy for osteonecrosis of the femoral head. In addition, Ito *et al*. [4] reported an 81% survival rate at 10 years, 60% at 20 years, and 50% at 25 years after femoral wedge osteotomy for developmental dysplasia of the hip joint.

THA after several kinds of osteotomy is accompanied by various technical difficulties [10, 11, 27-29]. However, the effects of differences in varus osteotomy procedures on surgical difficulties of THA are unclear. Standardizing the surgical technique and implant specifications is important for the comparison of results after THA among varus osteotomy procedures. Design of the hip prosthesis has varied among patients in most reports; however, we used the same cementless prosthesis design in all cases in the present study.

In our study, femoral subtrochanteric corrective osteotomy combined with THA was performed in four hips. The indication for femoral subtrochanteric osteotomy was a requirement to shorten the femur for a highly dislocated hip or to correct the shape of the femur for a deformed proximal femur [30]. Some papers have reported good clinical and radiographic results of THA combined with subtrochanteric osteotomy; however, this is a technically demanding treatment option with characteristic complications, such as pseudarthrosis and intraoperative fracture [29-31].

Notably, total JOA hip score of the Curved group at the latest follow-up was significantly higher than the score in the Wedge group. Subtrochanteric osteotomy may thus have reduced total JOA hip score in the Wedge group; however, we also noted significant differences in total JOA hip score between the two groups, even after excluding subtrochanteric osteotomy cases. This suggests that surgeons should choose curved varus osteotomy with the objective of obtaining better clinical results.

Regarding the stem position, there were no significant differences between the two groups; however, stems that deviated from normal range were all in the Wedge group. Wedge varus osteotomy may thus hamper accurate insertion of the stem into the femoral canal.

Several limitations were associated with the present study. First, the diseases that were indications of varus osteotomy remain unknown. Second, the study group was relatively small (18 hips). However, this is the first report comparing clinical and radiographic results between wedge and curved varus osteotomy. Third, the average follow-up period was 72 months, which is relatively brief. Further, investigations are needed to establish the clinical results in detail and to outline a more specific clinical therapeutic strategy.

## CONCLUSION

To our knowledge, this is the first comparison of clinical and radiographic results after THA following failed curved or wedge varus osteotomy. When surgeons perform femoral varus osteotomy, they should consider the possibility of future conversion to THA. The present findings suggest that curved varus osteotomy is more suitable than wedge osteotomy for future conversion to THA.

## Figures and Tables

**Table 1:** Demographic characteristics of the study participants.

–	Total	Wedge Group	Curved Group	P Value
Number of patients	15	12	3	
Number of hips	18	14	4	
Sex (male, female)	2, 16	2, 12	0, 4	0.043
Follow-up period, months (mean ± SD, range)	72.2 ± 11.1, 54-91	70.1 ± 11.6, 54-91	79.5 ± 5.0, 72-84	0.143
Body weight, kg (mean ± SD, range)	57.8 ± 15.3, 42.0-103.9	58.3 ± 16.7, 42-103.9	55.8 ± 10.8, 49.7-72.0	0.780
Body height, cm (mean ± SD, range)	152.7 ± 7.3, 144.0-172.3	152.6 ± 5.6, 144.0-163.0	152.9 ± 12.9, 145.7-172.3	0.969
BMI, kg/m^2^ (mean ± SD, range)	24.6 ± 5.0, 19.5-39.1	24.8 ± 5.7, 19.5-39.1	23.7 ± 0.7, 23.1-24.4	0.495
Age at THA, years (mean ± SD, range)	56 ± 11, 25-74	57.1 ± 11.8, 25-74	52.8 ± 0.5, 52-53	0.485
Age at osteotomy, years (mean ± SD, range)	27.1 ± 10.8, 10-46	27.0 ± 11.6, 10-46	27.3 ± 9.1, 14-35	0.969
Duration after osteotomy, years (mean ± SD, range)	29.1 ± 9.0, 11-51	30.1 ± 9.0, 11-51	25.5 ± 9.4, 17-39	0.386
Osteotomy at pelvic side, n (%)	2 (11%)	1(7%)	1(25%)	0.316
Remaining implants, n (%)	5 (28%)	5 (36%)	0(0%)	0.160
Crowe classification Grade 1	7	6	1	
Grade 2	6	4	2	
Grade 3	3	2	1	
Grade 4	2	2	0	

THA: total hip arthroplasty

SD: standard deviation

**Table 2:** Results of perioperative findings.

–	Total	Wedge Group	Curved Group	P Value
Number of patients	15	12	3	
Number of hips	18	14	4	
Operation time, min (mean ± SD, range)	70.5 ± 27.2 36-134	73.8 ± 29.1, 37-134	59.0 ± 17.0, 36-770	0.352
Total blood loss, g (mean ± SD, range)	817.8 ± 381.1 260-1620	776.4 ± 365.9, 260-1532	963.0 ± 454.7, 577-1620	0.404
Perioperative blood loss, g (mean ± SD, range)	422.8 ± 276.2 118-1040	396.4 ± 257.7, 118-842	515.5 ± 360.6, 220-1040	0.464
Postoperative blood loss, g (mean ± SD, range)	395.0 ± 284.5, 0-1030	380.0 ± 308.7, 0-1030	447.5 ± 202.4, 150-580	0.689
Subtrochanteric corrective osteotomy, n (%)	4 (22%)	4 (29%)	0 (0%)	0.160
Cup size, mm (mean ± SD, range)	48.8 ± 2.6, 46-58	48.3 ± 1.3, 46-50	50.5 ± 5.0, 48-58	0.443
Stem distal size, mm (mean ± SD, range)	11.1 ± 1.4, 9-14	11.0 ± 1.3, 9-13	11.5 ± 1.9, 10-14	0.548
Ball size, mm (mean ± SD, range)	28.2 ± 0.9 28-32	28.0 ± 0.0 28-28	29.0 ± 2.0 28-32	0.059
Number of screws (mean ± SD, range)	2.7 ± 1.0, 2-6	2.9 ± 1.1, 2-6	2.0 ± 0.0, 2-2	0.147

SD: standard deviation

**Table 3:** Clinical results.

–	Total	Wedge Group	Curved Group	P Value
Number of patients	15	12	3	
Number of hips	18	14	4	
JOA hip score before THA (mean ± SD, range)	45.1 ± 16.3, 26-77	46.1 ± 16.6, 26-77	41.5 ± 17.1, 27-60	0.630
Pain (mean ± SD, range)	15.3 ± 8.5, 0-35	15.4 ± 9.3, 0-35	15.0 ± 5.8, 10-22	0.943
Gait (mean ± SD, range)	8.9 ± 4.4, 0-15	8.9 ± 4.5, 0-15	8.8 ± 4.8, 5-15	0.945
ROM (mean ± SD, range)	9.6 ± 5.0, 1-18	10.4 ± 5.2, 1-18	6.8 ± 3.4, 4-11	0.209
ADL (mean ± SD, range)	11.4 ± 3.2, 6-18	11.5 ± 3.3, 6-18	11.0 ± 3.5, 8-14	0.792
JOA hip score at last follow-up (mean ± SD, range)	85.1 ± 10.9**, 62-96	82.5 ± 11.1**, 62-96	94.0 ± 2.8*, 90-96	P<0.01
Pain (mean ± SD, range)	35.3 ± 5.5, 20-40	34.3 ± 5.8, 20-40	38.8 ± 2.5, 35-40	0.162
Gait (mean ± SD, range)	16.4 ± 4.9, 5-20	15.4 ± 5.1, 5-20	20.0 ± 0.0, 20-20	P<0.01
ROM (mean ± SD, range)	15.6 ± 1.7, 12-18	15.2 ± 1.7, 12-16	16.8 ± 1.0, 16-18	0.110
ADL (mean ± SD, range)	17.8 ± 1.7, 16-20	17.6 ± 1.8, 16-20	18.5 ± 1.0, 18-20	0.340

THA: total hip arthroplasty

JOA: Japanese Orthopaedic Association

SD: standard deviation

ROM: range of motion

ADL: activity of daily living

*p<0.05, **p<0.01

**Table 4:** Radiographic evaluation.

–	Total	Wedge Group	Curved Group	P Value
Number of patients	15	12	3	
Number of hips	18	14	4	
Cup abduction, degrees (mean ± SD, range)	40.5 ± 5.0, 32-51	40.5 ± 2.3, 32-51	40.5 ± 4.0, 37-46	0.986
Cup anteversion, degrees (mean ± SD, range)	16.2 ± 6.5, 3.5-29.1	18.0 ± 5.8, 9.9-29.1	10.3 ± 5.9, 3.5-17.8	P<0.05
Number of cups in malposition, n (%)	3 (17%)	2 (14%)	1 (25%)	0.612
Number of stems in malposition (varus-valgus), n (%)	1 (6%)	1 (7%)	0 (0%)	0.582
Number of stems in malposition (flexion-extension), n (%)	3 (17%)	3 (21%)	0 (0%)	0.310

SD: standard deviation
